# Identification of a novel *METTL23* gene variant in a patient with an intellectual development disorder: a literature review and case report

**DOI:** 10.3389/fped.2024.1328063

**Published:** 2024-07-04

**Authors:** Jian Zha, Yong Chen, Fangfang Cao, Yanghong Yu, Ruiyan Wang, Jianmin Zhong

**Affiliations:** ^1^ Department of Neurology, Jiangxi Provincial Children’s Hospital, Nanchang, China; ^2^ Department of Radiology, Jiangxi Provincial Children’s Hospital, Nanchang, China

**Keywords:** *METTL23*, intellectual disability, developmental delay, magnetic resonance imaging, gene variant

## Abstract

*METTL23* belongs to a family of protein lysine methyltransferases that methylate non-histone proteins. Recently, the *METTL23* gene has been reported to be related to an intellectual developmental disorder, autosomal recessive 44. Patients present with developmental delay, intellectual disability (ID), and variable dysmorphic features. Here, we report on a Chinese girl who presented with global developmental delay, abnormal brain structure, and multiple facial deformities, including a short/upturned nose with a sunken bridge, thin lips, and flat occiput. Whole-exome sequencing identified a novel variant (NM_001080510.5: c.322+1del) on the *METTL23* gene. This variant was not collected on public human variants databases such as gnomAD, predicted to influence the splicing as a classical splicing variant, and classified as Pathogenic according to the American College of Medical Genetics and Genomics (ACMG) guidelines. Since patients with *METTL23*-related ID are rare, we summarize and compare the clinical phenotype of reported patients with *METTL23* variants. Our report further expands the *METTL23* variants and provides new evidence for clinical diagnosis of *METTL23*-related ID.

## Introduction

Intellectual disability (ID) is a frequently observed phenotype in clinical genetics, with an estimated prevalence of 1%–3% worldwide (1). It occurs independently or as a component within a more intricate neurological or systemic syndrome. It is believed that genetic factors contribute significantly to a substantial portion of ID patients (2). Transcription plays a pivotal role in the consolidation of memory and is essential for the establishment of long-term synaptic plasticity and memory retention. Recently, more and more transcriptional regulations were identified as ID genes (3).

*METTL23* encodes a transcription factor regulator, first identified as the pathogenic gene of ID (OMIM: 615942) from a large Yemen consanguineous family (4). After its initial identification, several variations of the gene were reported (5–8). The main clinical phenotypes include developmental delay in motor and/or language, seizures, specific facial features, abnormal brain MRI, and other behavioral problems, such as autism and attention-deficit hyperactivity disorder (ADHD).

Here, we identify a novel *METTL23* gene variant (NM_001080510.5: c.322+1del) in our patient. She presented with developmental delay in motor and language. Her white matter myelination might be delayed compared to children of the same age, and the splenium of the corpus callosum developed thinly. Our report summarizes and compares the clinical phenotypes of patients with *METTL23* gene variants and expands the spectrum of *METTL23* gene variants related to ID.

## Case report

Our patient is a girl from a consanguineous family aged 1 year 10 months who was born at full-term by normal delivery without an abnormal birth history or other family history of disease. She was able to lift her head at 4 months, roll over at 5 months, sit unassisted at 10 months, and presented developmental delay aged 1 year 3 months. When she was admitted to our hospital, she could not crawl or stand, her hands were not flexible in grasping objects, and she could only make “da, ma, ba” sounds.

**Figure 1:**
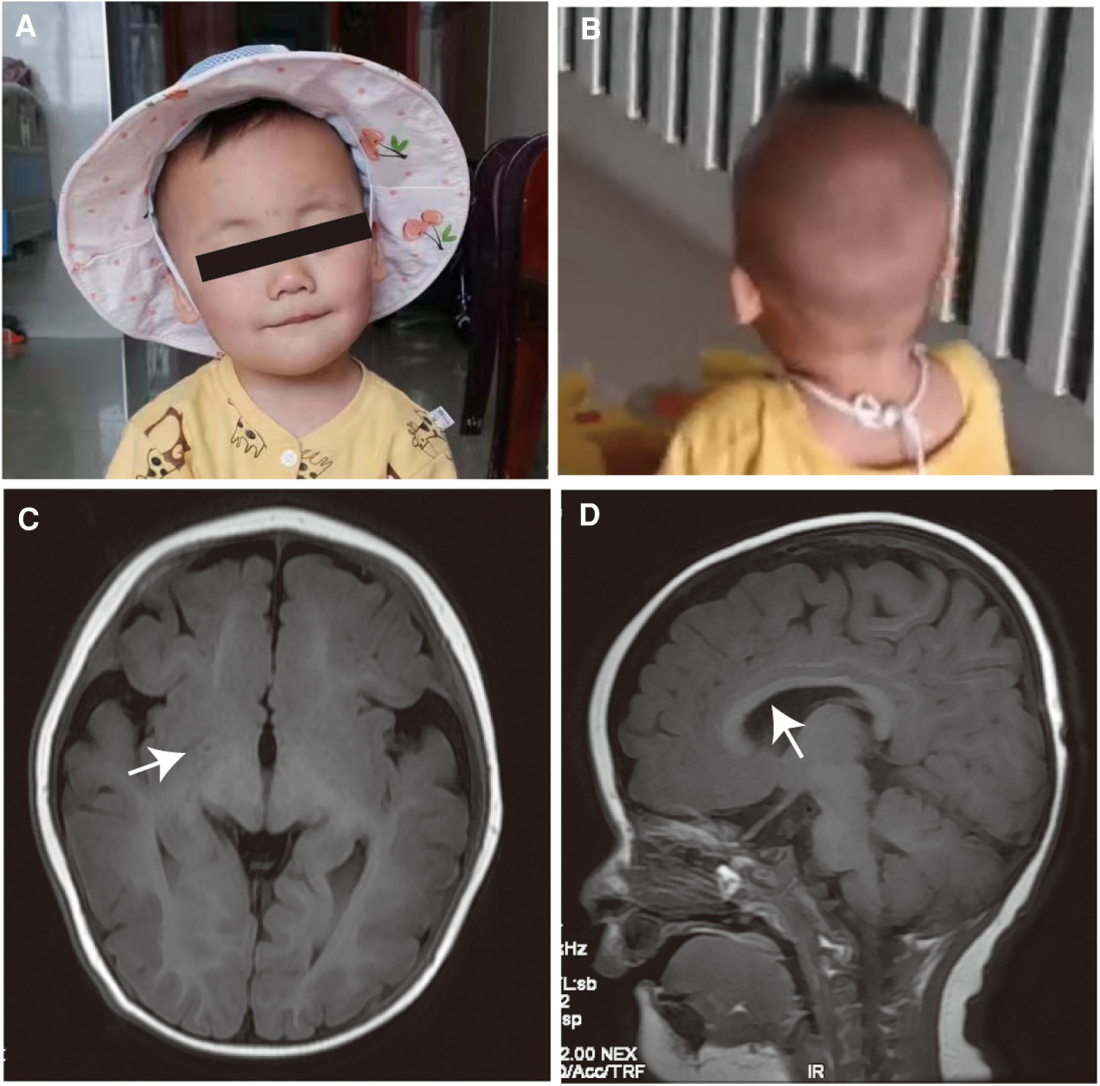
Clinical features. (**A,B**) Facial features of the affected individual. She has a short, upturned nose with a sunken bridge, thin lips, and flat occiput. (**C,D**) The brain MRI results in an infant aged 1 year 3 months. The white arrows show delayed white matter myelination and the thinly developed splenium of the corpus callosum.

She has specific facial features, with a short, upturned nose with a sunken bridge, thin lips, and a flat occiput (Figures 1A,B). The electroencephalography results showed that the background activity was slow, but no seizures were present. Brain MRI results suggested that white matter myelination might be delayed when compared with children of the same age, and the splenium of the corpus callosum developed thinly (Figures 1C,D). She has been undergoing rehabilitation training locally and has not returned to the clinic. During telephone follow-up, the child's development gradually improved. Currently, she can imitate adults’ speech, has learned to say goodbye, and learned body language, such as clapping and shaking her head. She is currently crawling steadily and can stand with support. We also incorporated previously reported *METTL23* gene variant cases in our analysis. Additional phenotypic and genetic findings in individuals are summarized in Table 1.

**Table 1:** Summary of the clinical features of patients with *METTL23* variants.

Ref	Patient ID	Gender	Age at last evaluation (years)	Consanguinity	Developmental delay (IQ)	Behavioral problems	Seizures	Specific facial features	Brain MRI	*METTL23* variant(s)
Our report	1	Female	1.3	−	NA, moderate	−	−	+	White matter myelination might be delayed in children of the same age, and the splenium of the corpus callosum develops thinly	Homozygous c.322+1del
Khan et al.	V-2	Female	10	+	NA, severe	Aggressive behavior	+	+	NA	Homozygous c.310T>C, p.Phe 104Leu
	V-4	Female	12	+	NA, severe	Aggressive behavior	+	+	NA	Homozygous c.310T>C, p.Phe 104Leu
	V-5	Female	24	+	NA, severe	Aggressive behavior	+	+	NA	Homozygous c.310T>C, p.Phe 104Leu
Smaili et al.	Patient 1	Male	7	+	NA, mild	−	−	+	NA	Homozygous c.176_177insG, p. Glu60Glyfs*11)
	Patient 2	Female	6	+	NA, mild	−	−	+	NA	Homozygous c.176_177insG, p. Glu60Glyfs*11)
Almannai et al.	Subject 1	Male	6	−	73	ADHD	−	+	Small and more vertically oriented left hippocampus compared to the normal appearing right one	Compound heterozygous c.470_471delTT, p.Leu157Rfs*4 and c.407+1G>C
	Subject 2	Female	3.5	+	NA	−	−	+	Not assessed	Homozygous c.322+2T>C
	Subject 3	Male	9	+	57	ADHD	+	+	Mild ventriculomegaly	Homozygous c.449T>C, p.Met150Thr
	Subject 4	Male	18	+	60	ADHD	−	+	NA	Homozygous c.322+2T>C
	Subject 5	Male	24	+	NA	ADHD	−	+	NA	Homozygousc.322+2T>C
	Subject 6	Male	13	+	58	ADHD	−	+	NA	Homozygousc.322+2T>C
Reiff et al.	IV-14	Male	26	+	NA, severe	Autism	+	+	NA	Homozygous c.169_172delCACT, p.His57Valfs*11
	IV-16	Male	18	+	NA, moderate	−	+	+	NA	Homozygous c.169_172delCACT, p.His57Valfs*11
	IV-19	Female	8	+	NA, moderate	−	−	+	NA (normal brain CT)	Homozygous c.169_172delCACT, p.His57Valfs*11
Bernkopf et al.	LFKK1 II:1	Female	54	−	NA, mild	−	−	−	NA (normal brain CT)	Homozygous c.281_285delAAGAT, p. Gln94Hisfs*6
	LFKK1 II:2	Male	52	−	NA, mild	−	−	−	NA	Homozygous c.281_285delAAGAT, p. Gln94Hisfs*6
	LFKK1 II:5	Male	43	−	NA, mild	−	−	−	NA	Homozygous c.281_285delAAGAT, p. Gln94Hisfs*6
	LFKK1 II:4	Female	39	−	NA, mild	−	−	−	NA	Homozygous c.281_285delAAGAT, p. Gln94Hisfs*6
	PK31 II:1	Female	NA	+	NA, mild	−	−	−	Increased volume of the subcallosal gray matter (less prominent compared to sibling PK31II:2)	Homozygous c.397C>T; p.Gln133*
	PK31 II:2	Female	NA	+	NA, mild	Aggressive behavior and disrupted impulse control	−	−	Increased volume of the subcallosal gray matter and decreased delineation of the basal ganglia region implicated in affecting regulation	Homozygous c.397C>T; p.Gln133*

## Genetic testing

**Figure 2:**
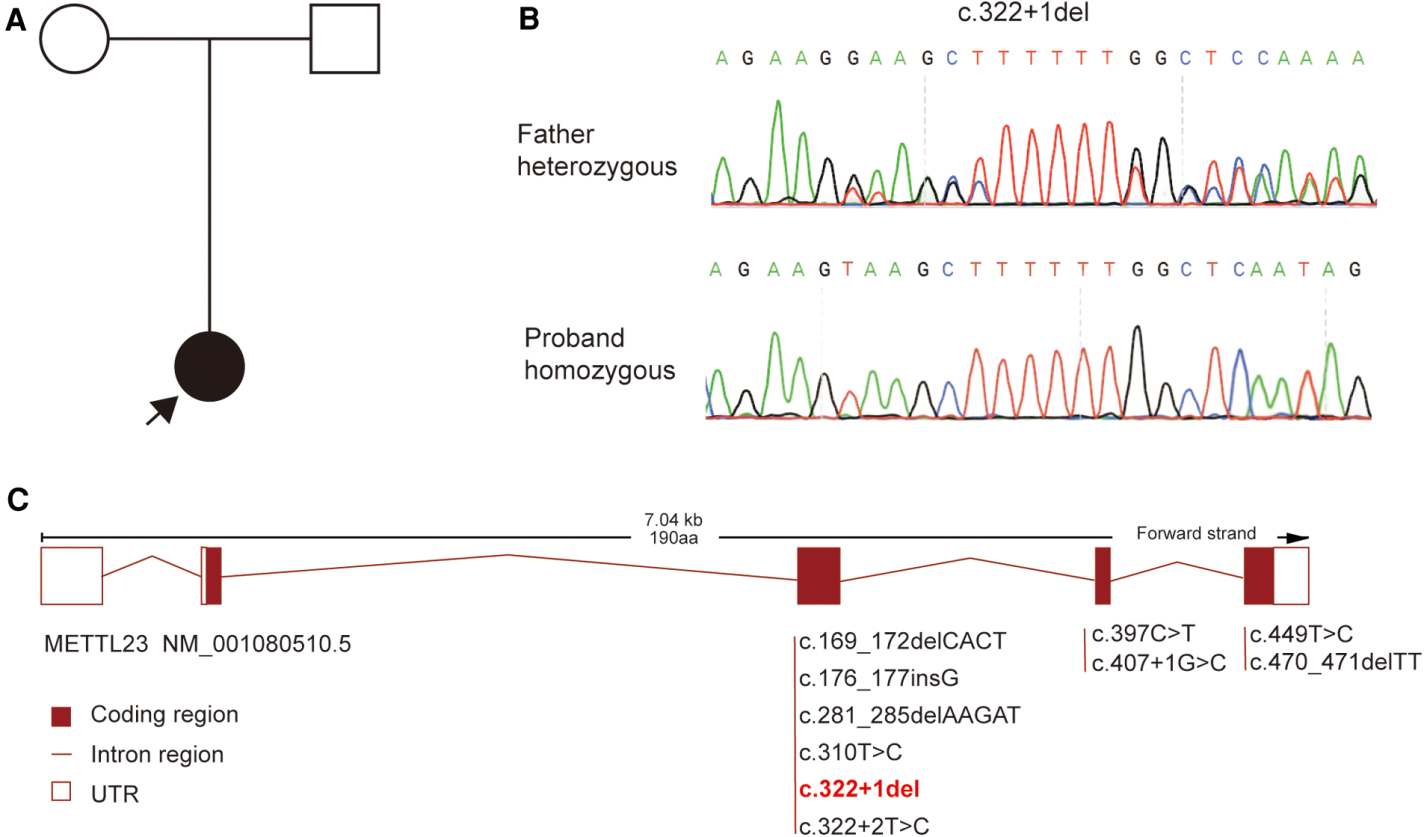
The genetic finding for our patient. (**A**) The pedigree for our patient. (**B**) Sanger sequencing verified the variant we found. (**C**) The spectrum of *METTL23* gene variants has been found.

Whole-exome sequencing was performed to further clarify the cause for our patient. A homozygous splicing variant of the *METTL23* gene (NM_001080510.5: c.322+1del) was identified by further genetic testing. The *METTL23* variant found in our patient was inherited from her father (Figure 2A), which was confirmed using Sanger sequencing (Figure 2B). The classical splicing variant c.322+1del has a Splice AI score >0.5 (Donor Loss score = 0.83). The *METTL23* variants were rare, and the variant in our patient was not included in gnomAD, ExAC, ClinVar, or other databases. Therefore, c.322+1del in our patient was classified as pathogenic according to the American College of Medical Genetics and Genomics guidelines (ACMG) (9) (PVS1+PM3_Supporting+PM2_Supporting).

## Discussion

The *METTL23* gene is deduced based on the *METTL22* sequence (10) and as a member of the non-histone methylating protein lysine methyltransferase family. It encodes a 190 (isoform 1) or 123 (isoform 2) amino acid protein, but the function of this gene is largely unknown. It was first reported as a pathogenic gene to ID in 2014, and it has a positive regulatory effect on GABP (GA-binding protein transcription factor) function through its interaction with the subunit GABPA (4). Genes regulated by GABP, such as THPO and ATP5B, may play roles in cognitive and neurodevelopment (4). THPO is thought to be involved in neuroprotection, apoptosis, development, and nerve cell differentiation (11), while ATP5B is downregulated in the thalamus of autistic patients (12). To further elucidate the catalytic performance of *METTL23*, Bernkopf et al. modeled the three-dimensional structure of human *METTL23* and clarified the methyltransferase function of *METTL23* (5).

With further exploration of the function of *METTL23* protein and the discovery of clinical cases, *METTL23* gene variants have been associated with an intellectual developmental disorder, autosomal recessive 44 (OMIM: 615942). Currently, there are 10 reported mutations in the *METTL23* gene, including our report. These mutations consist of two missense variants, five nonsense variants, and three splice site mutations. The mutation spectrum of the *METTL23* gene is primarily dominated by homozygous mutations, with only one instance of compound heterozygous mutations. The mutation in our patient is c.322+1del, which affects the classical splicing site. The *METTL23*-related disease is reported to be caused by loss of function (LOF) (5).

The main clinical features related to *METTL23* variants are developmental delay in motor and/or language and specific facial features. Patients with *METTL23* variants exhibit different levels of ID. Reiff et al. (4) reported their patient's ID was moderate to severe, but in the study by Bernkopf et al. (5), it was mild. The patient in our report showed mild ID. Her development gradually improved through rehabilitation training. We did not find an association between the severity of ID and the type of variant, and it may require additional functional verification to understand. Symptoms may include behavioral problems, such as ADHD, aggressive behavior, and seizures (6, 7). Neither behavioral problems nor seizures have occurred in our patient. She presented with developmental delay and specific facial features with a short, upturned nose with a sunken bridge, thin lips, and a flat occiput. This facial deformity exhibits a high level of consistency in previous reports. It confirms that *METTL23* may be a distinct clinical entity associating ID with specific facial dysmorphia (7). In addition, our patient had abnormal MRI results, which showed white matter myelination delay and thin splenium of the corpus callosum. This MRI abnormality was not reported previously, nor were small and more vertically oriented left hippocampus, mild ventriculomegaly (6), and increased volume of subcallosal gray matter (5). This indicates that the brain structural abnormalities caused by *METTL23* gene variants may be diverse.

In conclusion, we have summarized the clinical characteristics of all previously reported cases. Patients with developmental delay, varying degrees of intellectual disability, behavioral abnormalities, and epilepsy should undergo genetic testing. This will help clarify the cause of their condition and provide early clinical diagnosis and treatment. Our study contributes a new case of ID with *METTL23* variants, making this the first reported case in China. At present, the specific mechanism of *METTL23* mutation and the occurrence of ID are unknown, and more cases and further experiments are needed to understand the specific pathogenesis.

## Data Availability

The variant site presented in this study can be found in online repositories. The names of the repository/repositories and accession number(s) can be found here: https://www.ncbi.nlm.nih.gov/clinvar/variation/2571608/?oq=SCV003936901m=NM_015166.4(MLC1):c.838_843delinsATTTTA%20(p.Ser280_Phe281delinsIleLeu), SCV004041809.
